# Prismatic adaptation coupled with cognitive training as novel treatment for developmental dyslexia: a randomized controlled trial

**DOI:** 10.1038/s41598-024-57499-9

**Published:** 2024-03-26

**Authors:** Giulia Conte, Lauro Quadrana, Lilian Zotti, Agnese Di Garbo, Massimiliano Oliveri

**Affiliations:** 1 Child and Adolescent Neuropsychiatry Division, Department of Human Neuroscience, Sapienza University of Rome, 108 via dei Sabelli, 00185 Rome, Italy; 2 NeuroTeam Life & Science, 112 via della Libertà, 90143 Palermo, Italy; 3 Department of Biomedicine, Neurosciences and Advanced Diagnostics (BiND), University of Palermo, 129 via del Vespro, 90127 Palermo, Italy

**Keywords:** Health care, Medical research

## Abstract

Despite intense and costly treatments, developmental dyslexia (DD) often persists into adulthood. Several brain skills unrelated to speech sound processing (i.e., phonology), including the spatial distribution of visual attention, are abnormal in DD and may represent possible treatment targets. This study explores the efficacy in DD of rightward prismatic adaptation (rPA), a visuomotor adaptation technique that enables visuo-attentive recalibration through shifts in the visual field induced by prismatic goggles. A digital intervention of rPA plus cognitive training was delivered weekly over 10 weeks to adolescents with DD (aged 13–17) assigned either to treatment (N = 35) or waitlist (N = 35) group. Efficacy was evaluated by repeated measures MANOVA assessing changes in working memory index (WMI), processing speed index (PSI), text reading speed, and words/pseudowords reading accuracy. rPA treatment was significantly more effective than waitlist (p ≤ 0.001; ηp2 = 0.815). WMI, PSI, and reading speed increased in the intervention group only (p ≤ 0.001, ηp2 = 0.67; p ≤ 0.001, ηp2 = 0.58; p ≤ 0.001, ηp2 = 0.29, respectively). Although modest change was detected for words and pseudowords accuracy in the waitlist group only (words: p ≤ 0.001, d = 0.17, pseudowords: p = 0.028; d = 0.27), between-group differences were non-significant. rPA-coupled cognitive training enhances cognitive and reading abilities in adolescents with DD. This innovative approach could have implications for early remedial treatment.

## Introduction

Developmental dyslexia (DD) is a neurodevelopmental disorder characterized by poor reading acquisition, despite adequate intelligence and sensory-motor skills^1^. Across alphabetical languages, DD occurs approximately in 5–10% of the population^2^ and may present with variable impairment in reading fluency, reading accuracy, text comprehension, and spelling^1^. Etiology is multifactorial with mixed contribution of genetic and environmental factors^2–4^, and the disorder often co-occurs with other neurodevelopmental conditions, most prominently attention deficit/hyperactivity disorder (ADHD)^5^.

Several theories addressed the issue of the critical neurocognitive deficit underlying poor literacy. Decades of research have converged on the role of phonological deficits, positing that DD arises from a congenital impairment in phonological awareness (i.e., the ability to represent, store, and retrieve speech sounds) with consequent poor letter-sound correspondence^6–11^. An alternative approach to explain DD is the visual deficit theory^12,13^, which emphasizes the contribution of perceptual deficits to poor reading through abnormal visual coding of letters/words^14–18^. Integrations to the visual theory of DD have been later added by accumulating evidence of disruptions beyond simple visual processing, involving visual attention and temporal discrimination abilities^19–22^. Notably, a visual attention “preference” to the right visual hemifield with mild difficulties in orienting attention to the left side has been shown in children and adults with DD^23,24^. Hari et al.^25^ first proposed that impaired regulation of visual attention plays a central role in literacy development, by showing that dyslexic readers exhibit asymmetric distribution of visual attention with less efficient processing of visual stimuli from the left visual hemifield. This has lent support to the conceptualization of DD as a left-sided ‘minineglect’ syndrome^26,27^ hypothetically linked to a minor parietal lobe dysfunction because, in neglect syndrome, patients similarly disregard—although to a greater extent—the left side of their extra-personal space due to a contralateral parietal lobe damage. In addition to the visuo-attentive and phonological deficit theories, other valuable contributions to the understanding of DD come from the cognitive level, where deficient information-processing mechanisms have been outlined. Current theoretical explanations in this regard include deficits in general processing speed^28–31^, weaker verbal working memory (WM), planning, and response inhibition^32–34^. Overall, the available evidence does not allow to set a unifying theory of the neuropsychology of dyslexia, and a variety of cognitive manifestations exist in the disorder and relate to the actual network of impairment^35,36^.

Despite multiple neurocognitive models of DD, not much is available in terms of theory-based treatment approaches and evidence-based evaluation of their efficacy. Current treatments seem to be only partially effective and mostly train phonological or language abilities^37^. However, the lively investigation for the brain basis of DD beyond the phonological framework is promoting new treatment avenues aimed at enhancing reading abilities in indirect ways, that is, by training perceptual and cognitive skills apparently compromised in the disorder (e.g.^38,39^). Furthermore, there is a pressing need that new interventions not only improve reading performances, but also demand low levels of resources and result appealing to children^40–43^.

First implemented in neglect syndrome patients, prismatic adaptation (PA)—a rehabilitation technique that allows a visuo-motor recalibration towards the neglected visual hemifield^44^—might hold promising treatment potential for DD. PA uses prismatic goggles that displace the visual image rightwards or leftwards while the participant is asked to perform a visuomotor pointing task, causing an initial mismatch between the participant’s movement and the actual position of the target stimulus. However, when PA is repeatedly applied, the brain tries to correct this visual shift by realigning visual with proprioceptive ‘maps’, resulting in a visuo-motor recalibration in opposite direction to prism deviation that persists after the removal of the goggles, the so-called ‘prism after-effect’^45^. Interestingly, the prism after-effect is not simply a sensorimotor phenomenon but improves several more complex cognitive functions, likely through brain plasticity mechanisms (for a review, see^46^).

Several studies have particularly pointed out that, in neurotypical individuals, PA results in visual and proprioceptive realignment through the activation of the cerebellum, and temporo-parietal and posterior parietal cortices^47–50^. Given the central role of the right parietal cortex in visuo-spatial processing and attention^51–53^, these findings may further indicate that PA manipulates the visuo-spatial allocation of attention through the initial error signal generated by prism exposure. This is further supported by the evidence that in both patients with neglect as well as in healthy individuals, different lines of evidence support that PA enhances visual search abilities^54^, orienting of attention^55–57^, and spatial/temporal representations of stimuli^58–60^. More specifically, rightward-deviating lenses have shown to strengthen the activation of brain attentional networks^61–63^, while leftward-deviating lenses to improve phonological skills^64^. Moreover, unlike other complex visual stimulation techniques requiring extended exposure, adaptation to prisms quickly develops over the course of a 5-min simple pointing session^65,66^, thus ensuring good affordability and tolerability. Thus, PA has been proposed as a simple, non-invasive tool to induce neuroplasticity within visual attention networks^61,67^. This is of special clinical interest in DD for several reasons. First, dyslexia emerges during developmental ages, therefore there is great need to involve children and young adolescents in interventions that are realistically suitable for them, i.e., brief and undemanding, yet attractive. Second, since any rehabilitation technique needs to be carried out repeatedly to allow automatization through brain plasticity modifications, PA opens an effortless entry route to space representation and processing by acting on higher-level cognition in such a way as to bypass the conscious rehabilitation of visuo-attentive abilities^45^. Therefore, the bottom-up approach provided by PA might successfully target the abnormal attention orienting processes which have been shown in DD^26,68^.

Learning to read is a complex process that could be constrained by several cognitive factors, ranging from low-level sensory to higher level cognitive processes. Working memory—the individual’s capacity for retaining information for short term so as to allow its use or manipulation to carry out various cognitive tasks^69^—underpins important academic functions (e.g.^70,71^) and there is evidence supporting the idea that aspects of working memory are impaired in some but not all children with dyslexia^72–74^. Information processing speed is another cognitive ability that has been proposed to facilitate different higher order cognitive activities by allowing the simultaneous unfolding of multiple cognitive processes^75^. Dyslexics have been found to perform less well on a variety of temporal resolution tasks, including tests of visual attention, temporal judgement, and auditory discrimination and sequencing^68,76,77^, with research suggesting that processing speed is related, at least in a subset of dyslexic readers, to automaticity and fluency^78^. Therefore, working memory and processing speed do add a little variation to reading performance that is not explained by phonological abilities and linguistic comprehension^79^ and may represent a valuable additional target in remediating DD.

Hence, we addressed the possibility that DD might be partly remediated by acting on its visual attention imbalance through visuo-motor recalibration by means of PA, and that simultaneous training of working memory and processing speed skills may add greater and more sustained effects to PA intervention. Therefore, we set out to investigate the efficacy of a combined bottom-up (PA) and top-down (cognitive training) non-phonological intervention for DD. Treatment consisted of 10 weekly sessions of rPA treatment plus cognitive training and was delivered to adolescents with DD aged 13–17 years with a delayed intervention group serving as control. Given the above premises, we expected to observe two major findings: (i) an enhanced reading performance in the active intervention compared to the control group, mirroring both processes of optimized orientation of visual attention through PA as well as enhanced working memory/processing speed through cognitive training, and (ii) a consistent modulation of working memory and processing speed abilities in the treatment group.

## Materials and methods

### Participants

A total of 70 participants aged 13 to 17 years (mean ± SD: 15.8 ± 0.7 years) receiving a first diagnosis of DD were recruited from the Learning Disorders specialty clinic at the Child and Adolescent Neuropsychiatry Division, Sapienza University of Rome. The study was approved by the institutional Research Ethics Committee (Comitato Etico Lazio Area 1) and all participants, and their parents provided their informed written assent and consent to study participation, respectively. Diagnosis of DD was based on neuropsychological evaluation, according to DMS-5 criteria^1^ and conformed with the Italian criteria^80^, i.e., having a score below two standard deviation (− 2 SD) in at least 2 of 6 tasks assessing reading speed and accuracy. Participants were consecutively recruited from March 2022 to June 2022 and none had previously undergone treatments specifically targeted at improving literacy skills. Inclusion criteria were as follows: (a) diagnosis of DD; (b) total IQ above the low average range (≥ 80), as confirmed on the Wechsler intelligence scale for children—fourth edition (WISC-IV^81^); (c) either Working Memory Index (WMI) or Processing Speed Index (PSI) below the low average range (< 80), or both. The following exclusion criteria were considered: (a) presence of other neurodevelopmental disorders, particularly of attention-deficit/hyperactivity disorder (ADHD); (b) any major comorbid psychiatric disorder such as schizophrenia, bipolar disorder, or major depression disorder; (c) a diagnosis of active epilepsy; (d) physical disabilities that could impair the use of the study instruments. Type (a) and (b) exclusion criteria were evaluated through the Kaufman Schedule for Affective Disorders and Schizophrenia (KSADS-PL) clinical interview^82^ administered to the patient and to the caregivers, separately. Type (c) and (d) exclusion criteria were ruled out through accurate history taking by a child psychiatrist experienced in neuromotor and neurological disorders of childhood. The study has been publicly registered on 21/11/2023 on the ISRCTN registry (ISRCTN15190285). Consolidated Standards of Reporting Trials (CONSORT) guidelines were followed to report trial information and results^83^.

### Procedure

We conducted a single-center, two arm, randomized controlled PA trial. Participants were divided into two groups, with 35 subjects assigned to PA treatment group and 35 to Waitlist group. After clinical evaluation and diagnostic disclosure with the family, participants were randomized either to a 10-week intervention at no cost or compensation, or to a waitlist group. Group allocation was obtained through a stratified randomization method to control and balance the influence of covariates. Sex (two levels: male, female) and age (two levels: 13.0–14.9 years, 15.0–16.9 years) were used to achieve balance among groups in terms of participants' baseline characteristics (covariates). With these two covariates, possible block combinations total four (e.g., female in the 13.0–14.9 age range). Thus, the two groups were counterbalanced for sex (p = 0.81), and did not differ in chronological age, full intelligent quotient (IQ), WMI, and PSI (all p-values > 0.05). Further, the two groups did not differ in any pre-treatment reading ability (Table 1). A simple randomization procedure, such as flipping a coin, was used to assign participants within each block to one of the two treatment groups.

**Table 1:** Participants’ demographics and neuropsychological baseline characteristics.

	Treatment		Controls		Group comparison	
	Mean	SD	Mean	SD	Mann–Whitney U, *p*	χ^2^, *p*
Age	15.79	0.78	15.68	0.72	529.0 , 0.33	–
Male	15	–	17	–	–	0.230, 0.81
Female	20	–	18	–	–	0.230, 0.81
WMI	77.80	6.87	80.77	6.16	766.5, 0.07	–
PSI	78.37	8.40	78.89	6.17	670.0, 0.50	–
Reading speed	3.76	0.69	3.93	0.76	705.5, 0.27	–
Word reading accuracy	94.14	0.83	93.92	0.66	556.5, 0.51	–
Pseudoword reading accuracy	89.56	4.42	89.78	3.60	670.0, 0.50	–

*WMI* working memory index, *PSI* processing speed index.

Reading speed is expressed in syllables/seconds, while accuracy as percentage (%). p represents p values (two-tailed).

All participants allocated to the waitlist group were informed at baseline that they would be offered the intervention at post-test, i.e., after 10 weeks, but their outcomes after treatment were not included in the present study to keep possible confounding variables to a minimum. A power analysis was conducted to set sample numerosity with an estimated medium effect size, f, of 0.50, with correlations among repeated measure, r, of 0.7 and powered at 85% (G*Power software v3.1.9). According to power analysis, enrollment in the study was stopped upon reaching the target sample size of n = 35 for each study group. The intervention group received 10 weekly sessions of rightward PA (rPA) combined with computer-based cognitive training, each session lasting approximately 30 min (10 min for the rPA phase and 20 min for the cognitive training). All subjects were right-handed and had normal or corrected-to-normal vision. Testing was performed at baseline (max. 1 week before treatment started) and 10 weeks after the beginning of the treatment protocol (within max. 5 days after the last session). There are no potential harms known from the use of PA although potential tiredness due to treatment length (once-weekly sessions over 10 consecutive weeks) could not be excluded. However, none of the participants either experienced or reported any adverse event during the trial conduction.

### Treatment protocol

Participants in the intervention group were administered ten weekly sessions of rPA coupled with cognitive training delivered through the MindLenses software (www.restorativeneurotechnologies.com). The treatment protocol consisted of two phases. First, a visuo-motor adaptation task with use of prismatic goggles was performed by each participant, followed by tablet-delivered neurocognitive training. The training room was a dimly lit area organized to provide the fewest number of distraction sources, and views from the windows were blocked. Instructions for the task were verbally given to each participant by experimenters at the beginning of each session.

#### rPA task

During the first phase of each treatment session, participants were instructed to follow point with their right index finger at a target randomly located on a tablet screen while wearing 20-diopters rightward-deviating prismatic goggles. Task duration was 3 min corresponding to approximately 150 pointing movements^63^. Initially, the prisms cause displacement of the participant’s visual focus to 20° to the right of the physiological focus determining a pointing error in the direction of the prism deviation. After a few numbers of trials, participants spontaneously correct such calibration error and point correctly to the target, i.e., a visuo-motor recalibration occurs. Further details of the rPA task are available in Supplementary Materials.

#### ‘Serious games’ neurocognitive training

Immediately after rPA, participants performed seven ‘serious games’, i.e., tablet games which tap into attention, inhibition, working memory, and problem solving. Further details of the tasks are given in Supplementary Materials (see also^84^). By combining simulation, entertainment, and learning, serious games simulate specific activities to improve cognitive skills.

### Assessment materials

#### Wechsler intelligence scale for children-IV (WISC-IV)

The Italian version of the WISC-IV^85^ was applied to measure cognitive abilities (intelligence quotient, IQ). The WISC-IV contains 10 core subtests organized to provide four primary index scores (verbal comprehension, perceptual reasoning, working memory, and processing speed). Index quotients (M = 100, SD = 15) are calculated based on age norms. Working memory index (WMI) is one of the four indexes derived from the WISC-IV, which measures the ability to maintain focused attention, memorize and retain new information, and manipulate it to perform a task. Processing speed index (PSI) is another index derived from the WISC-IV measuring sustained attention, visual scan, discrimination, and retaining of simple-unknown visual stimuli in short-term memory, and visuo-motor coordination.

#### MT-Avanzate-3

It is an Italian test battery^86^ providing assessment of reading, writing and calculation abilities for students in the first 2 years of senior high school, and validated to diagnose specific learning disorders. Reading speed was assessed under the same viewing and luminance conditions for all subjects. Participants were asked to sit comfortably in front of a desk on which the reading card was placed at approximately 40 cm distance. Participants were instructed to read each text, loudly and as quickly as possible.

#### DDE-2 and MT 16–19

Either DDE-2^87^ or MT 16–19^88^ were used to assess accuracy and speed in reading single words and pseudo-words (pronounceable strings of letters with no meaning, generated by changing the characters of high-frequency words). DDE-2 and MT 16–19 were implemented for subjects under and over 16 years old, respectively. In the single word reading task, participants read out four lists of words with variable degree of complexity and frequency in the Italian language. This task measures the ability to recognize and correctly read known complex stimuli. In the pseudoword task, subjects read aloud three lists of pseudowords to assess the automatization of the grapheme-to-phoneme correspondence ability.

### Outcome measures

#### WMI and PSI

WMI and PSI were used to accurately evaluate differences between groups pre- and post-treatment, since they show little or no learning effects at re-test over time^89,90^.

#### Text reading speed

Text reading speed was implemented as main measure of reading automatization. It was assessed in syllables per second and calculated using this formula (syllables read correctly/seconds) by reading out loud the text included in the MT-Avanzate-3 battery.

#### Word and pseudoword reading accuracy

Single word and pseudoword reading accuracy were measured through the DDE-2 or MT 16–19 battery and both expressed in percentage values (100% value indicating maximum accuracy).

### Data analysis

Group differences at baseline were tested comparing all the pre-test measures with Mann–Whitney U tests for independent samples for continuous variables, and Chi-square tests for categorical variables. Treatment effects were examined with 2 × 2 (Group: treatment/waitlist × Time: pre-/post-test) repeated measures MANOVA. Effect sizes for all analyses (partial η^2^) are reported. As the value of η^2^ depends on the number and size of other effects in the model, partial η^2^ was considered as practical alternative^91^. Bonferroni-corrected post-hoc analyses were conducted to compare performances within the same group over time. Spearman’s correlations were computed between observed improvements in reading variables and WMI, and PSI, to evaluate relatedness of the outcome variables changes at post-test. All analyses were conducted with SPSS statistical software (V27.0; SPSS, Inc., Chicago, IL).

### Ethical standards

The authors assert that all procedures contributing to this work comply with the ethical standards of the relevant national and institutional committees on human experimentation and with the Helsinki Declaration of 1975, as revised in 2008. This study was approved by Comitato Etico Lazio Area 1 (155 viale del Policlinico, Rome, 00161, Italy), ref. n. 0195/2024.

## Results

### Treatment efficacy

**Figure 1:**
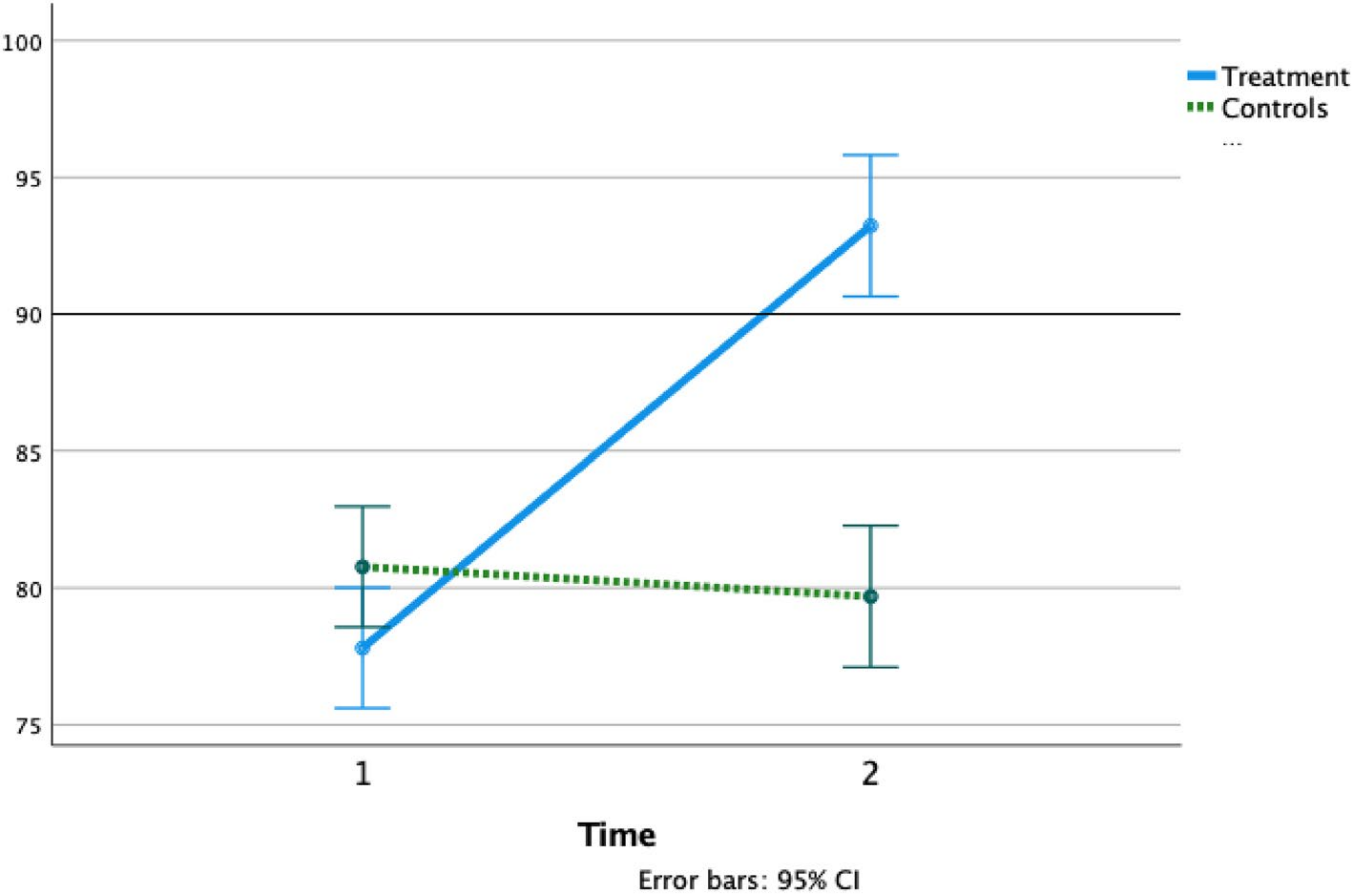
WMI mean change between groups from baseline to post-test. Working memory index (WMI) mean variation from baseline (1) to post-test (2) in the Treatment group (solid line) and Controls (dashed line).

**Figure 2:**
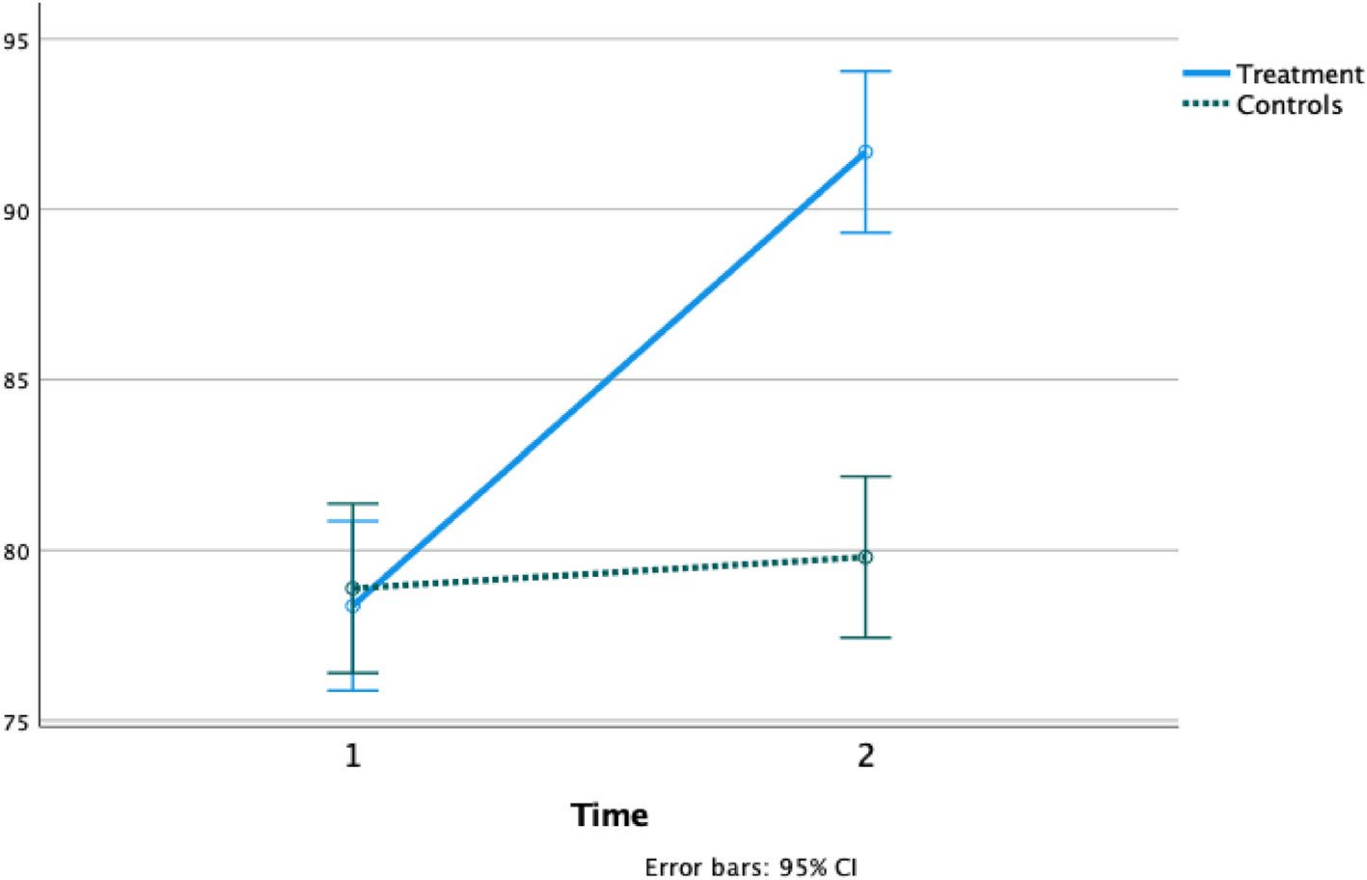
PSI change between groups from baseline to post-test. Processing speed index (PSI) mean variation from baseline (1) to post-test (2) in the Treatment group (solid line) and Controls (dashed line).

**Figure 3:**
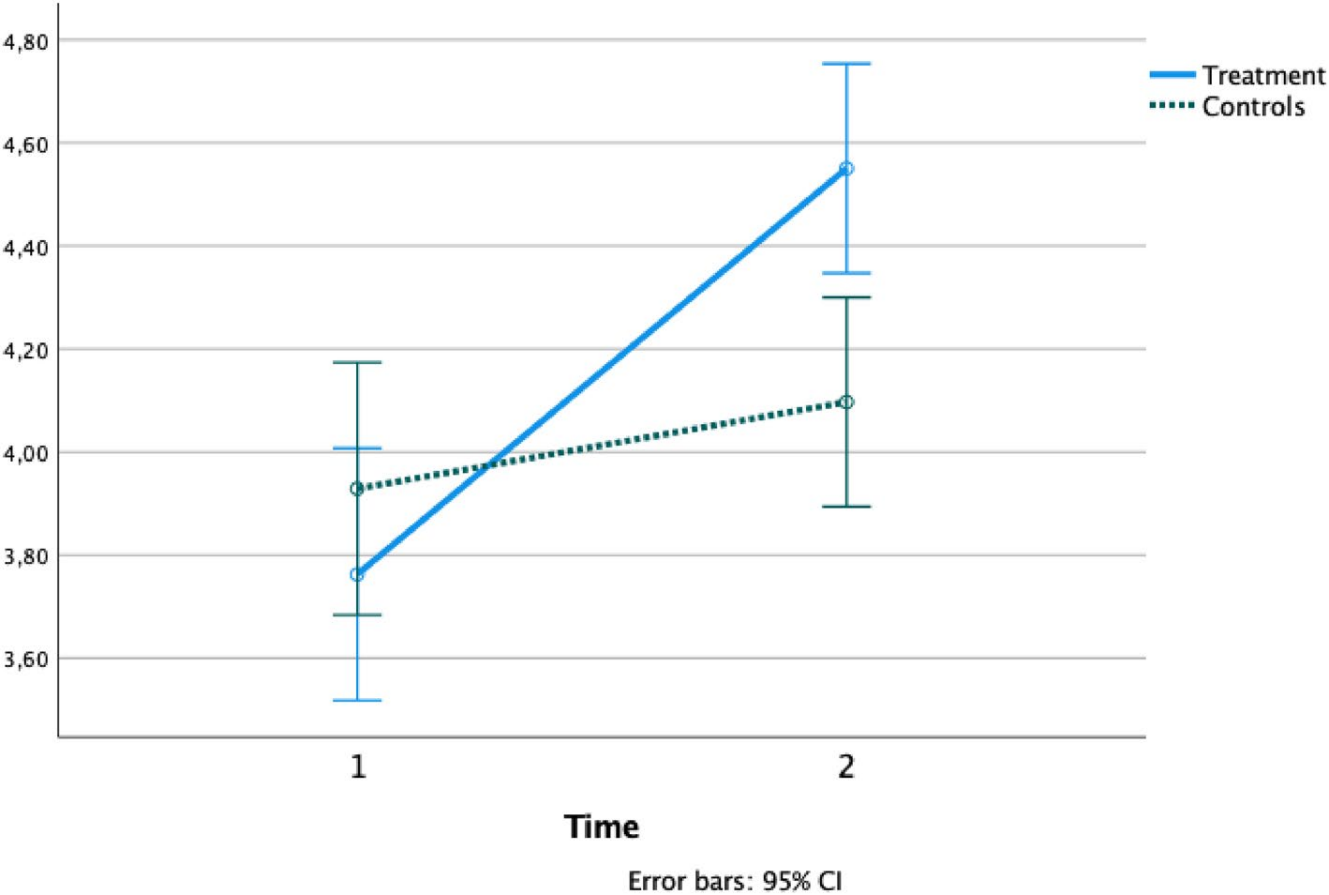
Text reading speed change between groups from baseline to post-test. Text reading speed mean variation from baseline (1) to post-test (2) in the Treatment group (solid line) and Controls (dashed line). Reading speed is presented as mean and expressed in syllables/second.

Data from all the 70 participants in the trial were included in the analyses. A 2 × 2 (PA group/Waitlist group × pre-/post-treatment) MANOVA with repeated measures revealed a significant effect of Time [F(7,62) = 89.456, p ≤ 0.001, η_p_^2^ = 0.910] on the outcome measures, indicating an overall increase in all outcome measures at post-treatment assessments. This effect was driven by a significant interaction effect for Group by Time [F(7, 62) = 38.990; p ≤ 0.001; η_p_^2^ = 0.815], revealing that changes in outcome measures over time were largely explained by group membership. Mean changes in WMI, PSI, and text reading speed from baseline to post-test assessment between groups are displayed in Figs. 1, 2 and 3.

At univariate level (Table 2), significantly better performances were found in the PA group for the following variables: WMI [F(1,68) = 132.313, p ≤ 0.001, η_p_^2^ = 0.67], PSI [F(1,68) = 91.257, p ≤ 0.001, η_p_^2^ = 0.58] and text reading speed [F(1,68) = 27.052, p ≤ 0.001, η_p_^2^ = 0.29]. No significant effect of treatment was found in word reading accuracy [F(1,68) = 3.62, p = 0.061] and pseudoword reading accuracy [F(1,68) = 0.078, p = 0.78] at group comparisons over time.

**Table 2:** Mean change in outcome variables from pre- to post-test between treatment and control groups.

	Group	Mean_1_ (SD)	Mean_2_ (SD)	F	p value	η_p_^2^
WMI	Treatment	77.74 (6.87)	93.25 (8.99)	132.313	** < 0.001**	0.67
	Controls	80.77 (6.16)	79.66 (6.05)			
PSI	Treatment	78.47 (8.40)	91.83 (6.79)	91.257	** < 0.001**	0.58
	Controls	78.89 (6.17)	79.66 (7.24)			
Text reading speed	Treatment	3.77 (0.69)	4.56 (0.66)	27.052	** < 0.001**	0.29
	Controls	3.93 (0.76)	4.09 (0.54)			
Word reading accuracy	Treatment	94.13 (0.83)	94.86 (0.94)	03.623	0.061	–
	Controls	93.92 (0.67)	94.94 (0.78)			
Non-word reading accuracy	Treatment	89.50 (4.42)	91.32 (4.97)	0.078	0.78	–
	Controls	89.78 (3.61)	92.05 (5.07)			

*WMI* working memory index, *PSI* processing speed index, *η*_*p*_^*2*^ partial eta squared, *Mean*_*1*_ mean pre-treatment, *Mean*_*2*_ mean post-treatment, *SD* standard deviation.

Bold font indicates a significant p-value (< 0.05).

### Treatment performance

Post hoc analysis showed that all outcome measures significantly increased from pre- to post-test in the PA group with moderate to large effect sizes, except for non-word reading accuracy (Table 3, Supplementary Figs. 2–4). Specifically, there was significant increase in WMI (p < 0.001, Cohen’s d = 1.22, 95% CI [13.13–17.72]), in PSI (p < 0.001, Cohen’s d = 1.01, 95% CI [11.78–14.85]), text reading speed (p < 0.001, Cohen’s d = 0.13, 95% CI [0.65–0.92]), and word reading accuracy (p < 0.003, Cohen’s d = 0.12, 95% CI [0.43–1.05]). Differently, in the control group, there was significant variation, although with small effect size, from pre- to post-test in the accuracy of word reading (p < 0.001, Cohen’s d = 0.17, 95% CI [0.91–1.08]), and pseudoword reading (p = 0.028; d = 0.27, 95% CI [0.27–4.33]), whereas no differences emerged in WMI, PSI, and text reading speed. At post-test, it could be observed that text reading speed improvements correlated to PSI (ρ = 0.392, p = 0.038), and PSI positively correlated with WMI (ρ = 0.352, p = 0.02) (Table 4).

**Table 3:** Mean change in outcome variables from pre- to post-test within treatment and control groups.

	Mean change	SD	t	p value (two-tailed)	Cohen’s d
Treatment					
WMI	15.43	6.68	13.658	** < 0.001**	1.22
PSI	13.31	4.46	17.645	** < 0.001**	1.09
Text reading	0.79	0.39	11.994	** < 0.001**	0.13
Word accuracy	0.74	0.91	4.888	** < 0.001**	0.12
Pseudoword accuracy	1.73	6.19	1.659	0.106	n.s.
Controls					
WMI	− 1.09	5.52	− 1.164	0.252	n.s.
PSI	0.91	6.51	0.831	0.412	n.s.
Text reading	0.17	0.58	1.07	0.097	n.s.
Word accuracy	0.99	0.24	24.382	** < 0.001**	0.17
Pseudoword accuracy	2.3	5.91	2.301	**0.028**	0.27

*WMI* working memory index, *PSI* processing speed index, *n.s.* non-significant.

Bold font indicates a significant p-value (< 0.05).

**Table 4:**
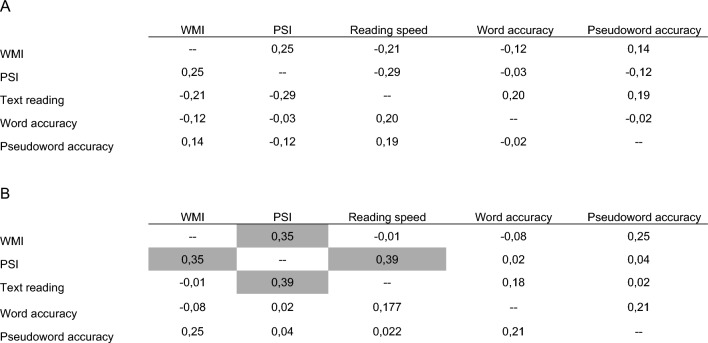
Correlations of cognitive outcome measures with reading abilities in the intervention group before (A) and after (B) treatment with controlling IQ.

Cells highlighted in gray represent correlations with *p* < .05.

## Discussion

To our knowledge, this is the first study to evaluate the efficacy of PA coupled with cognitive training for the treatment of DD. Our results demonstrate that 10 weekly sessions of the proposed intervention improved reading speed in the treatment group only, while it remained unvaried in the control group. Reading accuracy showed significant pre- to post-treatment improvements within both groups, specifically as per word spelling. However, differences in reading accuracy between groups were statistically non-significant. Furthermore, significant improvements in working memory and speed processing abilities were induced by treatment, with processing speed showing medium correlational strength to reading speed, therefore suggesting that the treatment allows modulation of cognitive functions relevant for the reading performance.

Research in dyslexia remediation has recently shifted emphasis from phonological to non-phonological interventions, to directly improve reading by acting on specific cognitive or perceptual skills that underlie this complex ability. Visuo-attentional approaches have proved particularly effective in this regard, improving literacy generally equal to or greater than ‘traditional’ interventions^92^. Visuo-attentional trainings for DD appear to be especially effective in boosting reading speed^40,93,94^. Our results are consistent with such findings, showing that working on attentional abilities contributes to greater reading speed in the Italian language. Concerning the type of visuo-attentional training, a few studies have implemented a lateralized treatment approach by stimulating primarily one visual hemifield, and showed efficacy in reading speed improvement as compared to other phonological trainings commonly used^95,96^. PA is a technique capable of inducing significant influence on the hemispheric lateralization of complex brain functions such as attention^62,63,97^. Namely, a model posits that prismatic adaptation increases the excitability of the hemisphere ipsilateral to the visual field deviation^59^. According to this model, rightward prismatic adaptation in the present study could have activated right hemispheric networks, thus counteracting the left mini-neglect, consistent with the hypothesis of Hari et al.^26^. On the other hand, another not mutually exclusive explanation, posits that after brief rPA exposure, an enhanced activation of the left parietal cortex within nodes of the attentional network is observed, while underactivation of the contralateral brain is obtained^61,101^. Thus, rPA could shift the hemispheric dominance for spatial attention from the right to the left hemisphere^62,63^. This shift toward modality-specific hemispheric representations may also be critical for DD remediation. In the human brain, robust structural and functional evidence supports that attentional networks show relative lateralization toward the right hemisphere^102,103^. This asymmetric hemispheric engagement in attentional control may be arguably even more pronounced in DD, since metanalytical evidence has pointed out reduced activation of large left brain networks in the disorder, particularly concerning the key nodes of the ventral attentional network^104–106^. Based on our findings, we suggest that rPA may boost reading fluency by acting as a lateralized stimulation that recalibrates attentional resources to the left ‘neglected’ visual hemifield, thus likely enhancing the visual processing of graphemes. This possibility awaits future studies that may confirm how rPA influences reading outcomes by analyzing neural correlates of attentional resource allocation following PA.

Based on the observed correlations, it could be hypothesized that the examined intervention further acts on reading speed, by boosting increase in processing speed rather than accuracy of performance. Several prior studies have explored the efficacy of working memory and executive functioning training in DD, by showing significant improvements in both central executive functioning as well as in reading fluency^93,107–109^. Our study expands upon this evidence by outlining that working memory and general processing speed abilities show significant change after intervention and moderately correlate with reading speed change. Of note, working memory and processing speed improved to a greater extent at post-treatment as compared to reading speed. This result suggests that executive function training is relevant for reading performance, at least in those individuals with DD that show poor working memory and cognitive speed. Further, reading speed is generally harder to remediate than accuracy deficits even after efficient intervention^110^ and extremely slow phonological decoding has been proposed as the core deficit in DD readers across both shallow and deep orthographies^111,112^. Supposedly, the enhancement of visuo-attentive skills may not only be induced by potentiating hemispheric attentional processes through rPA^44,45,99,113^, but it also may require visual information to be elaborated in a very short amount of time, to be retained, and manipulated. Interventions aimed at increasing working memory and processing speed may likely result in a greater degree of automatization of the component processes involved in the complex visual or visuo-verbal task of reading. Therefore, by possibly increasing the retention of multiple letter-sound correspondences and the execution rate of different higher-order cognitive functions, working memory and processing speed appear to add significant variation to reading performance and may well represent targets for DD treatment. Thus, based on our results, we support the notion that it may be useful to combine a hemisphere-specific stimulation such as PA with interventions targeting other transversal cognitive factors allegedly underpinning DD to provide greater effectiveness to DD remediation strategies.

We did not observe clinical dropouts in our study. Such high compliance to treatment suggests that the digital rPA-cognitive treatment program was feasible and sufficiently appealing to adolescents with DD, comparing favorably to previous other non-phonological interventions^93,114^. Moreover, the child-friendly digital interface of the proposed training with embedded game elements in the tasks, makes this intervention also suitable for younger children, thus, holding relevant potential for early DD remediation. Our protocol also implements an adaptive mechanism that constantly assesses the participant’s performance, and accordingly adapts the task difficulty. Thus, the software’s algorithm provides a dynamic modification of the task level, i.e., both providing increased difficulty when the performance is good as well as decreasing difficulty if the ongoing performance is weaker for that task in the specific session trained. This mechanism appears to be crucial to ensure good engagement in the task with low risk of frustration and motivation drop. This is a crucial aspect since adherence to treatment and time-saving interventions on targeted abilities are of utmost importance when analyzing the actual efficiency of training on reading skills, particularly in developmental ages.

### Limitations and future studies

Given that participants were not blinded to treatment because of the clear group allocation in our methodology, a selection bias is acknowledged as principal limitation of the study. However, perfect blinding is unfortunately rarely achieved in drug trials^115^, not to mention in behavioral treatment trials, where there is simply no way to create two outwardly identical interventions. A growing body of literature has been debating on the malleability of cognitive functions according to expectations (i.e., how much the single participant places a positive value on the intervention and expects it will improve a certain ability)^116,117^. However, significant debate still exists in the field with mixed results^118–122^. Crucially, some people respond to expectations whereas others do not^123^. Given that certain individual characteristics (e.g., personality, motivation) moderate the size of the expectation effect, future work should more carefully examine such interindividual differences^124^*.* Nevertheless, blinding patients to the type of treatment received in a controlled trial has proved particularly relevant when the response criteria are subjective, but less important for objective criteria^125^ such as reading speed or standardized assessments. Moreover, employing a no-treatment condition may be of particular use when investigating gains in abilities across developmental ages, to control for potential confounding factors such as spontaneous reading development and/or test–retest effects. A previous study on the efficacy of a reading intervention in Italian children^126^, employing the same reading tests as the present one readministered after 4 weeks, included a no-treatment condition, and reported no substantial improvement on the reading outcomes in this group. In our study, only modest improvement was seen in word and pseudoword reading accuracy in the control group (word: p < 0.001, Cohen’s d = 0.17, pseudoword: p = 0.028; d = 0.27), while no difference emerged in these outcomes between treatment and control groups from baseline to post-test. This likely suggests the presence of little spontaneous gains in reading accuracy within our sample, while the improvements after treatment in the other outcome measures should be considered sufficiently reliable in reflecting an actual change in ability.

We acknowledge as a critical study limitation the unfeasibility of separating the independent effects of PA against serious games on cognitive and reading outcomes. However, the study protocol was undertaken as such based on the evidence that patients with enduring post-stroke problems in executive functioning improved with PA plus serious games treatment only but not when receiving serious games training alone^84^. Moreover, PA and serious games have been further combined because they differently intervene on attentional processes. While PA is a bottom-up technique that allows a stimulus-driven recalibration of visual attention without requiring any volitional control, the serious games imply voluntary and conscious allocation of attentional resources. Thus, the two elements of the treatment differently engage attentional networks, with PA being processed by the ventral attention system^127,128^—involved in detecting unattended or unexpected stimuli and triggering shifts of attention^129^, and serious games stimulating top-down attentional processes that enable the participant to selectively choose where to focus based on expectations and conscious goals. Although requiring further investigation, our hypothesis is that each technique alone may result in a smaller influence on reading skills as compared to the combination of the two, which synergically act on two different routes of attention.

A further limitation of the study is the lack of experimental measures specifically tapping the attentional allocation through left or right hemispheric activation that are hypothesized to mediate the change in reading ability, which does not allow to test the actual brain dynamics of PA in improving reading. Future studies exploring neurophysiological and neuroimaging correlates of PA in DD remediation are therefore warranted to address this issue.

Moreover, our study did not involve any follow-up measurement after a significant period, thus preventing from making inferences regarding the duration of treatment effects. Future research should replicate these results with a follow-up after 6 months or at 1 year, to verify their long-term persistence. Finally, generalization of results should also be cautious. All participants included in our study had either one or both indexes of processing speed and working memory below the low average range (< 80) as assessed by the WISC-IV. This criterion was chosen to maximize treatment effects following cognitive training but may limit generalizability of our results. Future studies should allow to identify which students with DD may benefit more of the treatment by including dyslexic adolescents with more variable cognitive profiles, particularly with normal or above normal WMI and PSI.

## Conclusions

Our study highlights that PA combined with cognitive training enhances cognitive skills and reading speed in adolescents with DD. According to our initial hypothesis, PA coupled with cognitive training appears to foster reading fluency by optimizing the defective visual attention orientation of DD through PA as well as by strengthening executive functions. We suggest that this very combination of visuo-attentive recalibration and cognitive enhancement was the key to the effectiveness of the proposed intervention.

Our findings extend the previous suggestion of a left minineglect in dyslexic individuals^26^, and further provide interesting indirect information on the mechanisms involved in the disorder. By showing that reading performance improves in dyslexic adolescents following rPA, we suggest that disruption in lateralized visuo-attentional networks may be central to the pathophysiology of DD and that interventions specifically targeted at reverting this imbalance may reduce core difficulties.

Lastly, our findings expand knowledge on non-phonological remediation interventions in DD and provide additional evidence that attention and executive functions training may enhance treatment efficacy.

### Supplementary Information

Supplementary Information.

## Data Availability

The datasets used and/or analysed during the current study are available from the corresponding author on reasonable request.
