# Supplementary material for: Prismatic adaptation coupled with cognitive training as novel treatment for developmental dyslexia: a randomized controlled trial
Source: Sci Rep. 2024 Mar 26;14:7148. doi: 10.1038/s41598-024-57499-9 (PMC10965979; doi:10.1038/s41598-024-57499-9)
Supplement: Supplementary file 1 — Supplementary Information. [file 41598_2024_57499_MOESM1_ESM.docx]

**Prismatic adaptation coupled with cognitive training as novel treatment for Developmental Dyslexia: a randomized controlled trial**

**Supplementary Materials**

**PART 1 – Details on the Prismatic Adaptation (PA) protocol**

**rPA task**

The first phase of each treatment session consisted in a rightward PA (rPA) visuomotor training delivered by means of a pointing task. The pointing task consisted of a total of 150 trials and was subdivided in three main stages: preexposure, exposure, and postexposure (Supplemental figure 1).

1. “*Pre-exposure*”: here the participant is asked to tap with the right index finger on a visual target (black square, 2cm x 2cm size) randomly appearing on a tablet screen (30 trials). This step aims at enabling participants to become familiar with the task.
2. “*Exposure*”: participants perform the same task of the pre-exposure stage while wearing 20°-rightward-deviating lenses. During the exposure phase, participants can always see the trajectory of their movement (visible pointing) and are asked to point 90 times to targets. The prisms induce a 20° shift of the visual field to the right. This causes a mismatch between the participant’s pointing position and the target’s actual position, resulting in a pointing error in the direction of the prismatic deviation. This perceptual error quickly activates an automatic correction by the visuomotor system, i.e., the shift is corrected by realigning visual with proprioceptive ’maps’ ^1^. Therefore, in the late exposure phase, recalibration takes place, and the participant is now able to point with increased precision to the actual target position.
3. “*Post-exposure*”: the participant is again asked to indicate and touch the targets that randomly appear on the screen after the removal of the prismatic lenses (30 trials). In this phase, it is possible to observe the after-effect phenomenon, i.e., a leftward visuo-motor recalibration, consisting of a deviation to the left of the pointing spot compared to the actual position of the target. This was verified by asking the participant to look (without the prisms) at the target, then mark with eyes closed the supposed position of the target on the screen with their finger. The prism after-effect is interpreted as proxy of plastic reorganization of visuo-motor and visuo-attentional systems ^1–3^.

**Supplemental figure 1.** PA setup and timeline *- adapted from Bracco et al., 2018*. Participants point to targets on a tablet screen (here depicted as a curved surface, although the screen surface was flat). The dashed line marks the visual target position on the screen, while the arrow the pointing spot indicated by the participant. ***Pre-exposure*** (prismatic goggles off) involves pointing in free viewing conditions (both pointing movements and targets visible). During ***Exposure***, participants wear the googles (rightward orientation) and perform free viewing pointing (exposure, goggles on). In the early exposure phase, prismatic goggles determine a rightward shift of the visual focus, causing a pointing error (pointing spot marked to the left of the actual target position); in the late exposure phase, the pointing error is progressively resolved through visuo-motor recalibration. In ***post-exposure*** adaptation is tested immediately after exposure with blinded pointing to targets, consisting of a leftward displacement of the pointing spot compared to the target’s actual position (after-effect).


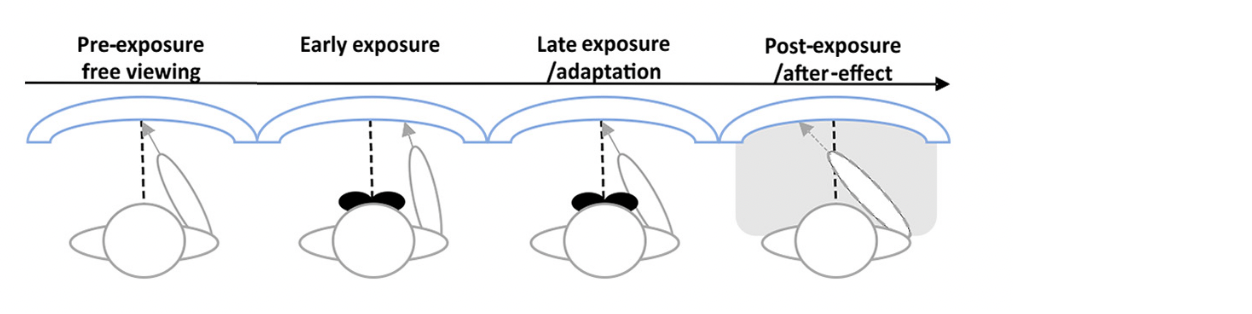


^4^

**“Serious games” neurocognitive training**

The second phase of each treatment session consisted of 7 serious games, delivered on a tablet to the participant immediately after the rPA protocol.

The proposed games have a pre-established time limit and a score (MindLenses ^TM^ [www.restorativeneurotechnologies.com](http://www.restorativeneurotechnologies.com)) (Supplemental Table 1). Each game contains an adaptive difficulty mechanism according to which the software increases or decreases the difficulty (on a scale of four levels) depending on the participant's performance. The score is calibrated according to the level of difficulty as well.

A brief explanation of the games included in the treatment protocol is provided below, with the exact order of the games within the protocol being represented in Supplemental Table 1. For more extensive details, a comprehensive description of serious games and all cognitive functions trained by each is also available in the work by Oliveri et al., 2023 ^5^.

Two of the games prosed pertain directly to the area of attentive functions:

1. "*Watch the bomb*" is a game consisting of touching the required target stimuli that flow at different speeds on the screen in the target area. This game improves the ability to keep attention to multiple stimuli for a prolonged time and inhibit interfering information (sustained attention).
2. "*The Café*" is a game consisting of remembering and preparing orders in a virtual coffee shop. It improves the ability to pay attention to multiple stimuli, and plan coherent actions (divided attention)

Two different games are related to the ability of interference control:

1. “*Are they the same?*” is a game consisting of tapping the target area on the screen only when two identical stimuli are presented, or if they are different but remain fixed. It enhances the ability to maintain sustained attention and inhibit competing responses (Go No-Go).
2. “*Which one doesn’t belong?*” is a game consisting of selecting the only shape different in form, color, or texture within a group of different visual targets. It improves the ability of perception and interference control (Visual search).

One serious game tap into the area of language:

1. “*Semantic association*” is a game consisting of associating professional figures with their work tools. It improves the skills of reasoning and making semantic associations.

Lastly, two serious games are related to working memory abilities:

1. “*Mathematical reasoning*” is a game consisting of completing basic operations, keeping the result in mind, and continuing to perform other operations. It improves the ability to calculate and manipulate information.
2. “*In reverse order*” is a game consisting of reordering the target images in reverse order of how they were presented before. It improves short-term memory and the ability to manipulate information. It pertains to the cognitive area of executive functions.

| **Cognitive Area** | **Test** | **Time limit (sec)** | **Game-over** | **Score** |
| --- | --- | --- | --- | --- |
| ***Attention*** | 1. Sustained attention ("*Watch the bomb*") | Variable (depending on performance: 10 lives assigned at the beginning) | Lives | Number of stimuli identified before game-over |
|  | 1. Divided attention ("*The Café* ”) | 120 | Time | % correct stimuli * multiplier |
| ***Interference control*** | 1. Visual search (“*Which one doesn't belong?*”) | 90 | Time | % correct stimuli * multiplier |
| ***Language*** | 1. *Semantic association (“The Jobs”)*” | 120 | Time | % correct stimuli * multiplier |
| ***Interference control*** | 1. Go no-Go (“*Are they the same?*”) | 90 | Time | % correct stimuli * multiplier |
| ***Working memory*** | 1. *“In reverse order”* | 120 | Time | % correct stimuli * multiplier |
|  | 1. “*Mathematical reasoning*” | 90 | Time | % correct stimuli * multiplier |

**PART 2 – Supplemental Figures**

Group median change of working memory index (WMI) and processing speed index (PSI) from pre- to post-treatment

* flags p values <.05

** flags p values <.01

*** flags p values <.001


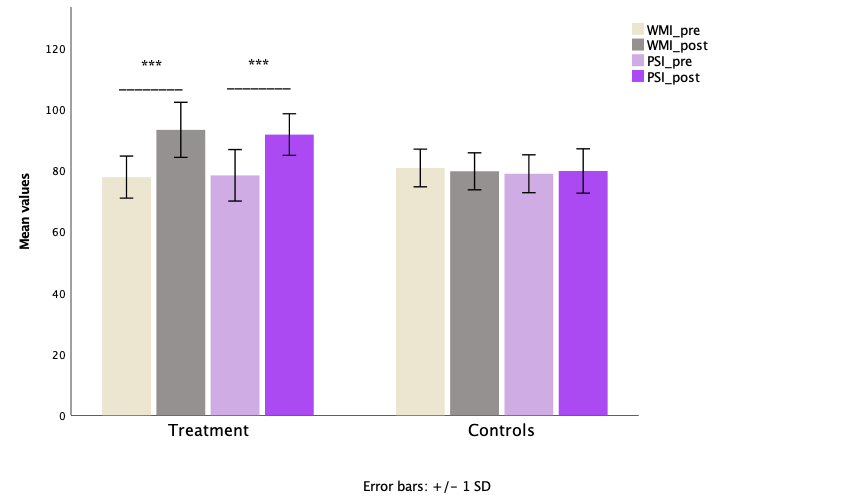


**Supplemental figure 2.** WMI and PSI median change within groups from pre- to post-treatment

Group median change of text reading speed expressed in syllables/second from pre- to post-treatment

* flags p values <.05

** flags p values <.01

*** flags p values <.001


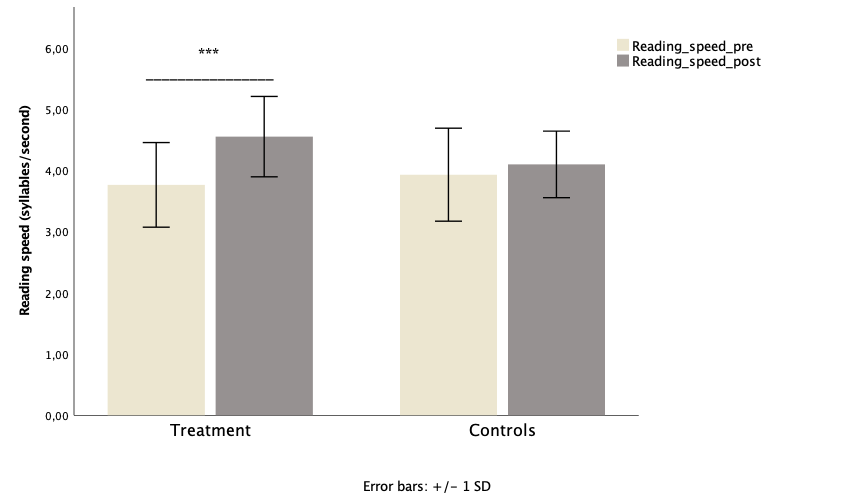


**Supplemental figure 3.** Text reading speed change within groups

Group median change in word and pseudoword reading accuracy expressed in percentage from pre- to post-treatment.

* flags p values <.05

** flags p values <.01

*** flags p values <.001

**Supplemental figure 4.** Word and pseudoword reading accuracy within groups


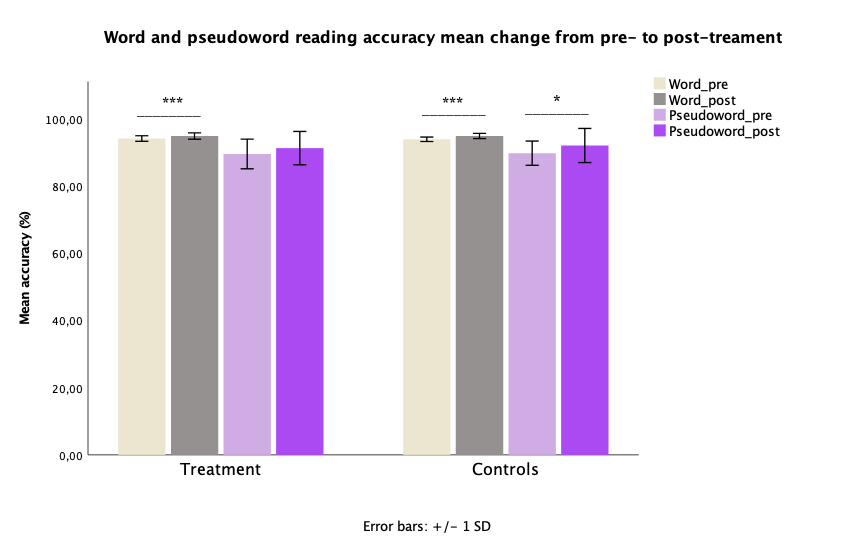


**References**

1. Pisella, L., Rode, G., Farnè, A., Tilikete, C. & Rossetti, Y. Prism adaptation in the rehabilitation of patients with visuo-spatial cognitive disorders. *Curr. Opin. Neurol.* **19**, 534–542 (2006).

2. Jacquin-Courtois, S. *et al.* Rehabilitation of spatial neglect by prism adaptation: a peculiar expansion of sensorimotor after-effects to spatial cognition. *Neurosci. Biobehav. Rev.* **37**, 594–609 (2013).

3. Michel, C. Beyond the Sensorimotor Plasticity: Cognitive Expansion of Prism Adaptation in Healthy Individuals. *Front. Psychol.* **6**, (2016).

4. Bracco, M., Veniero, D., Oliveri, M. & Thut, G. Prismatic Adaptation Modulates Oscillatory EEG Correlates of Motor Preparation but Not Visual Attention in Healthy Participants. *J. Neurosci.* **38**, 1189–1201 (2018).

5. Oliveri, M. *et al.* A novel digital approach for post-stroke cognitive deficits: a pilot study. *Restor. Neurol. Neurosci.* **41**, 103–113 (2023).
